# Assessing awareness to aid action: Stakeholders’ perception of invasive alien species in a metropolis from the Global South

**DOI:** 10.1038/s41598-026-59488-6

**Published:** 2026-08-26

**Authors:** Kesang C. Bhutia, Anzar A. Khuroo, N. A. Aravind

**Affiliations:** 1 Manipal Academy of Higher Education, Manipal, India; 2 Ashoka Trust for Research in Ecology and the Environment, S M Sehgal Foundation for Biodiversity and Conservation, Bengaluru, India; 3 Centre for Biodiversity & Taxonomy, Department of Botany, University of Kashmir, Srinagar, India

**Keywords:** Invasive Alien Species, Invasive species awareness, Global South, Ornamental plants, Urban area, Gardening, Ecology, Ecology, Environmental sciences, Environmental social sciences

## Abstract

Invasive alien species (IAS) introduced through the ornamental plant trade pose significant ecological threats globally, yet public awareness remains poorly studied in the Global South. Since awareness of IAS supports management efforts and encourages behavioral change, understanding knowledge gaps among key stakeholders is essential. Using the Knowledge-Attitudes-Practices (KAP) framework, this study evaluates IAS awareness among ornamental plant buyers and sellers in Bengaluru, India; one of the largest and fastest-growing metropolises in the Global South. Through structured interviews across 44 nurseries, we assessed IAS knowledge, factors influencing awareness, purchasing behaviors, and disposal practices that may contribute to IAS proliferation. Our findings reveal critically low awareness: fewer than 30% of buyers and only 5% of sellers were familiar with IAS concepts, and over 75% of respondents lacked knowledge of plant origins. Invasive species were significantly less recognised than native or non-invasive alien counterparts. However, when informed about the impacts of IAS, 89% of buyers expressed willingness to choose safer plant alternatives, and 57% of sellers indicated they would communicate risks to customers. These results highlight substantial knowledge gaps among urban horticultural stakeholders and underscore the transformative potential of targeted education. Bridging these gaps is critical for integrating IAS management into urban planning and biodiversity conservation across rapidly growing cities in the Global South.

**Supplementary Information:**

The online version contains supplementary material available at 10.1038/s41598-026-59488-6.

## Introduction

 Each year, approximately 200 new alien species establish themselves globally^1^, many arriving as ornamental species valued for their beauty but posing a severe ecological threat. Invasive alien species (IAS) are key drivers of global biodiversity loss and pose major environmental, economic, and human health risks^2,3^. The ornamental floriculture industry is a principal global pathway for these species^4^; yet remains insufficiently studied in the Global South. *Lantana camara*, among the world’s 100 worst invasive species^5^, was introduced to India as an ornamental in 1807, and has since become highly invasive^6^. Other examples include: *Ipomoea cairica*, *Mirabilis jalapa*, *Passiflora foetida*, *Ruellia tuberosa*, *Senna alata*^7^.

The ornamental trade generates multibillion-dollar revenues and provides livelihoods globally, but this economic success comes at a biosecurity cost. The sector facilitates rapid, high-volume trade of species, often prioritising aesthetic appeal over risk assessment^8,9^. While effective management of IAS requires coordinated action across multiple fronts from policy development to early detection and rapid response, the role of public awareness in preventing introductions and facilitating control measures has increasingly been recognised as foundational to successful management outcomes^10,11^.

In general, informed citizens are more likely to support invasive species management actively^12^. For instance, in South Africa, 63% of respondents who were initially unaware of invasive species expressed interest in learning more about the issue after participating in the study^13^. Similarly, in Portugal, those familiar with the “Invasive Plants in Portugal” program showed greater awareness and support for species removal^14^. Public understanding of invasive species not only facilitates cooperative management but also leads to successful education campaigns that encourage public involvement in prevention efforts^15^, ultimately fostering more environmentally responsible behavior^16^. Given the alarming rate of new species introductions worldwide, systematic assessments of public awareness are urgently needed to address this growing challenge effectively.

Despite growing recognition of the importance of the human dimension in biological invasions, systematic assessments of public knowledge and attitudes toward IAS, particularly within the ornamental plant trade sector, remain sparse, especially in the Global South. Research on IAS awareness is predominantly concentrated in the Global North (North America, Oceania, and Europe), accounting for approximately 69% of all studies^17^, reflecting a broader pattern in ecological research^18^. This geographic bias in the invasion biology literature is concerning, especially as emerging economies are predicted to face a surge in trade volumes and higher rates of species introductions^7^. Developing countries face distinct challenges in the prevention and management of biological invasions, including limited response capacity, insufficient stakeholder coordination, heavy reliance on biological resources, and low public awareness^7^. These structural vulnerabilities make understanding and addressing knowledge gaps in the Global South not merely an academic exercise, but an urgent conservation priority.

In the Global South, India exemplifies this emerging environmental concern. As one of the world’s largest flower-growing countries^19^, India is also a major importer of ornamental plants. Data from Trading Economics reports imports of live plants and cut flowers totalling US$54.15 million in 2024^20^. This trade volume, combined with India’s reliance on flowers, raises concerns about the spread of invasive species into vulnerable ecosystems. About 66% of India’s natural areas are at risk from invasive species, with plant invasions already affecting 22%^21^. The ornamental plant industry not only introduces potentially invasive species but also promotes known invaders, highlighting a lack of awareness among Indian stakeholders^7^.

Bengaluru, a rapidly growing metropolis in southern India, is known for its Information Technology (IT) hubs, parks, botanical gardens, institutes, bungalows with extensive gardens, and tree-lined roads^22^. Often called the ‘Garden City of India,’ Bengaluru’s rapid urban development, thriving ornamental plant trade, and growing invasion concerns make it an appropriate study area for examining the intersection of horticultural practices and invasive species spread. In this context, IAS awareness among ornamental plant buyers and sellers in Bengaluru was assessed using the Knowledge-Attitude-Practice (KAP) framework. The KAP model suggests that knowledge shapes attitudes, which in turn influence behaviour^23,24^. Here, we test three hypotheses: H1 examined current practices, focusing on aesthetic and cultivation-driven selection; H2 investigated knowledge gaps, specifically low awareness of IAS concepts and plant origins; and H3 evaluated whether providing knowledge could shift attitudes and behavioural intentions. By integrating these findings with the KAP framework, this study offers a case study from the Global South and contributes to the literature on invasive species awareness and management.

## Methods

### Study area

**Fig. 1:**
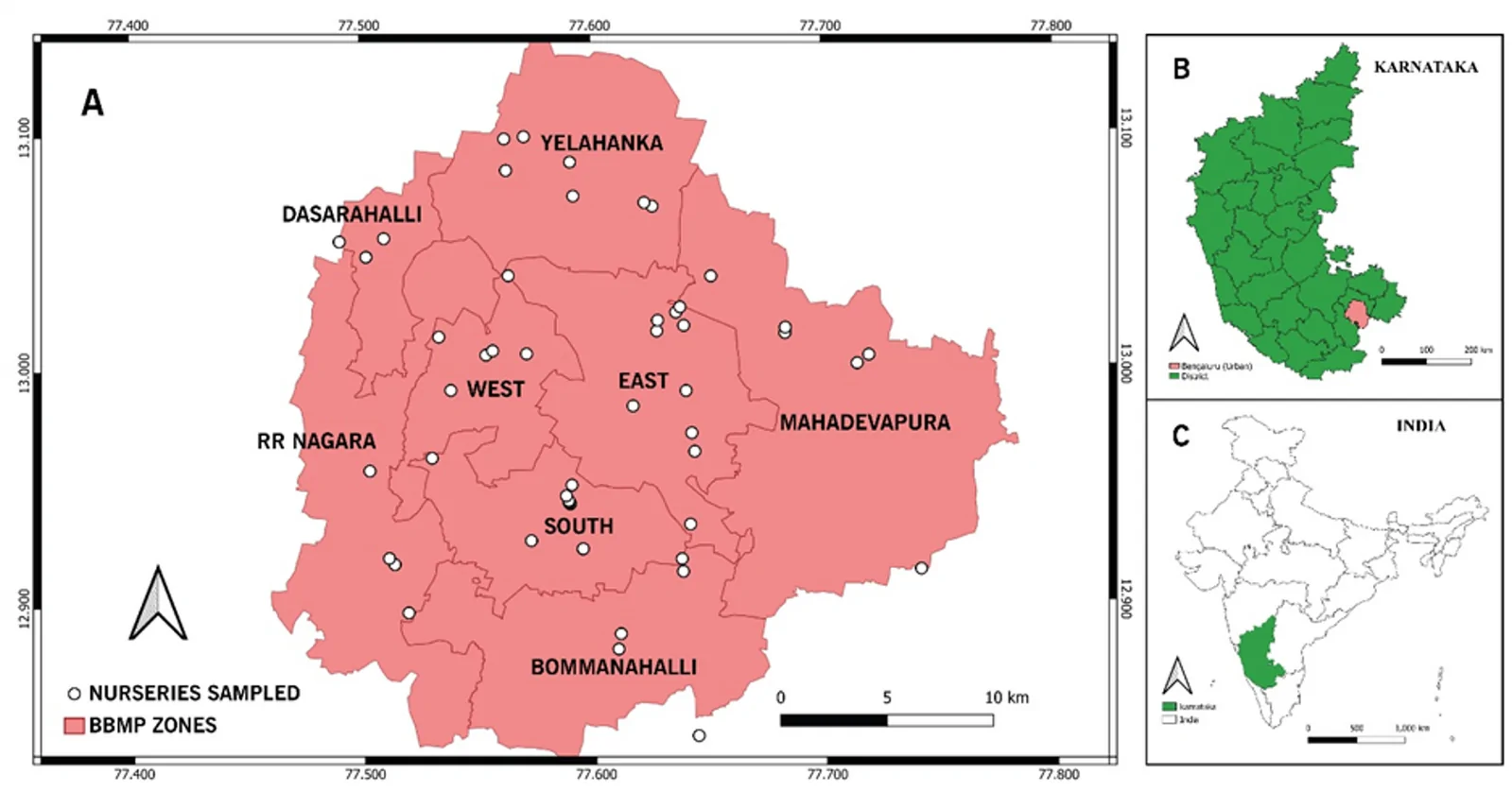
Map showing the eight administrative zones of Bengaluru with locations of the sampled nurseries (**A**). Inset maps show the location of Bengaluru within the state of Karnataka (**B**) and the location of Karnataka in India (**C**).

Bengaluru, the capital of Karnataka in southern India, lies between 12°39′00″–13°13′00″ N and 77°22′00″–77°52′00″ E at 920 m elevation on the Deccan Plateau^25^. During this study, the city was administered by the Bruhat Bengaluru Mahanagara Palike (BBMP) across eight zones (Fig. 1); the BBMP was subsequently replaced by the Greater Bengaluru Authority (GBA) following the implementation of the Greater Bengaluru Governance Act, 2024 (effective 15 May 2025). The South, West, and East zones form the historic core with traditional houses and large gardens. In contrast, IT-dominated Mahadevapura and Bommanahalli feature landscaped corporate campuses and apartment complexes, while the expanding Yelahanka zone, residential Rajarajeshwari Nagar zone, and development of the Dasarahalli zone contribute to the city’s dynamic landscape.

Bengaluru boasts extensive green cover across numerous parks, lakes, forested areas, cantonments, and large campuses associated with both public and private institutions^26^. The city is recognised as one of the hotspots for the introduction of alien plants in the country, with its own plant quarantine station and a central integrated pest management centre. Lalbagh, one of India’s oldest botanical gardens, was established by Hyder Ali in 1760 AD and later managed by British superintendents. It became a hub for introducing plants from across the globe. The Lalbagh Nursery, under Karnataka State’s Department of Horticulture, is a major wholesale nursery in Bangalore (https://horticulturedir.karnataka.gov.in/page/Gardens/Lalbagh/en; accessed 22/01/2024). Many nurseries benefit from Lalbagh’s plant materials and expertise^27^.

### Questionnaire surveys

In this study, respondents included both sellers and buyers of ornamental plants. ‘Buyers’ were defined as nursery customers, gardeners and landscapers while ‘sellers’ referred to nursery owners and employees. A stratified sampling method was used to select at least two large (> 10,000 plants) and two small (< 10,000 plants) nurseries per administrative zone (Fig. 1), totaling 44 nurseries across the city. Large nurseries cater to a wider market, including landscapers, with diverse plant selections and potentially higher prices, whereas small nurseries cater mainly to local customers, offering specialized or native plants at lower costs. Structured interviews were conducted with buyers and sellers (Supplementary material: A and B). The questionnaire was developed drawing on validated instruments from prior IAS perception studies^13,28–31^ and reviewed for content validity by the research team. As the survey comprised primarily binary, open-ended, and photo-identification questions rather than Likert-scale attitudinal items, internal consistency metrics such as Cronbach’s alpha were not applicable and are therefore not reported. The first section of the survey gathered demographic information including age, residence, and education. The respondents could optionally provide contact details to receive the published paper resulting from this study.

Buyers were asked about the types of plants they cultivated/bought and the reasons they chose them. Sellers identified the most popular plants and why they were in demand. Both groups were questioned regarding plant disposal practices, which may influence the spread of invasive species.

The photo-elicitation^29^ was used to aid plant identification; by showing respondents a collage (Supplementary material: C) of native and introduced ornamental plants in India and at Bengaluru nurseries. They were asked about plant origins (native or introduced), understanding of the term ‘invasive alien species,’ and awareness of the ecological impacts of invasive plants. The study also examined whether knowledge of invasive alien species could influence respondents’ behaviour. The questionnaire was the same for both groups, except that sellers were asked an additional question about their knowledge of invasive alien species and their willingness to inform customers.

### Data collection

The number of buyers surveyed in each nursery was determined based on the volume of daily customer traffic. At least 10% of the average daily number of customers were randomly selected and interviewed. All surveys for a given nursery were conducted on the same day for small nurseries or across 2–4 days for large nurseries. The average duration of each interview was approximately 9 min. The data were collected from July 2022 to March 2023.

In the case of sellers, interviews were conducted with at least one individual (employee or owner) in small nurseries and a minimum of two individuals (employees or owners) in larger nurseries. In the context of larger commercial nurseries, key personnel such as managers or chairpersons were also interviewed. In addition to the in-person surveys conducted, the questionnaire was also circulated as a Google Form with an introductory paragraph indicating the purpose of the study to increase the total number of respondents surveyed. The online survey was conducted from December 2022 and to June 2023. Owing to the similarity of the questions in the in-person and online questionnaires, data from the online surveys and in-person interviews were pooled for all analyses.

Finally, to raise awareness about IAS, each respondent was given a booklet at the end of the interview that included detailed information about invasive plants and their environmental impacts (Supplementary material: D and E).

### Data analysis

To explore the relationship between knowledge of IAS and factors such as gender, age, education level (no education, high school, diploma, graduate, postgraduate, and PhD), and gardening experience in years (< 2, 2–5, 5–10, 10–15, 15–20, and > 20), a logistic regression analysis was performed. Respondents unsure or unable to identify IAS were categorised as having ‘no’ awareness of IAS. Those who could describe IAS correctly, even with uncertainty, were placed in the ‘yes’ category. The independent variables included age, gender, gardening experience, and highest qualification. Model averaging was employed to address model uncertainty, with models having a ΔAICc of > 2 being averaged for robust parameter estimates. A chi-square test of independence was conducted to examine the association between stakeholder type (buyers and sellers) and awareness levels regarding invasive species.

We assessed respondents’ knowledge of plant origins shown in the photo collage. Respondents unsure of or unable to identify native regions were classified under ‘no’ awareness of plant nativity. Those able to identify native regions of some plants despite uncertainty were categorised under ‘yes’ for awareness of origins. A Z-test was conducted to assess buyers’ ability to identify invasive alien species compared to alien non-invasive and native species. We used the Jaccard similarity index to determine similarity among plants sold in different city zones. Statistical analyses, graphical representations, and word-cloud visualisations were performed using R version 4.3.3^32^. The text-mining package “tm” and word-cloud-generator package “word cloud” were used for text analysis and word-cloud generation. Logistic regression analysis was conducted using the “MuMIn” package to identify factors influencing awareness of invasive plants, and graphs were created using the ggplot2 package.

### Ethics statement

The research was granted ethical approval by the Institutional Review Board (IRB) of ATREE (IRB/ACA/0027/KB/01/2022), and all ethical guidelines were adhered to throughout the study. Prior to data collection, participants were thoroughly informed about the research objectives, emphasising that the findings would be utilised solely for academic purposes, with no commercial interests involved. Verbal consent was obtained from participants, allowing them to make informed decisions regarding their participation in the study, and their anonymity was assured. Participants who indicated a desire to obtain a copy of the published paper willingly provided their email addresses when asked. Demographic information of participants was collected but reported only in aggregate form to prevent individual identification.

## Results

### Demographic details

**Fig. 2:**
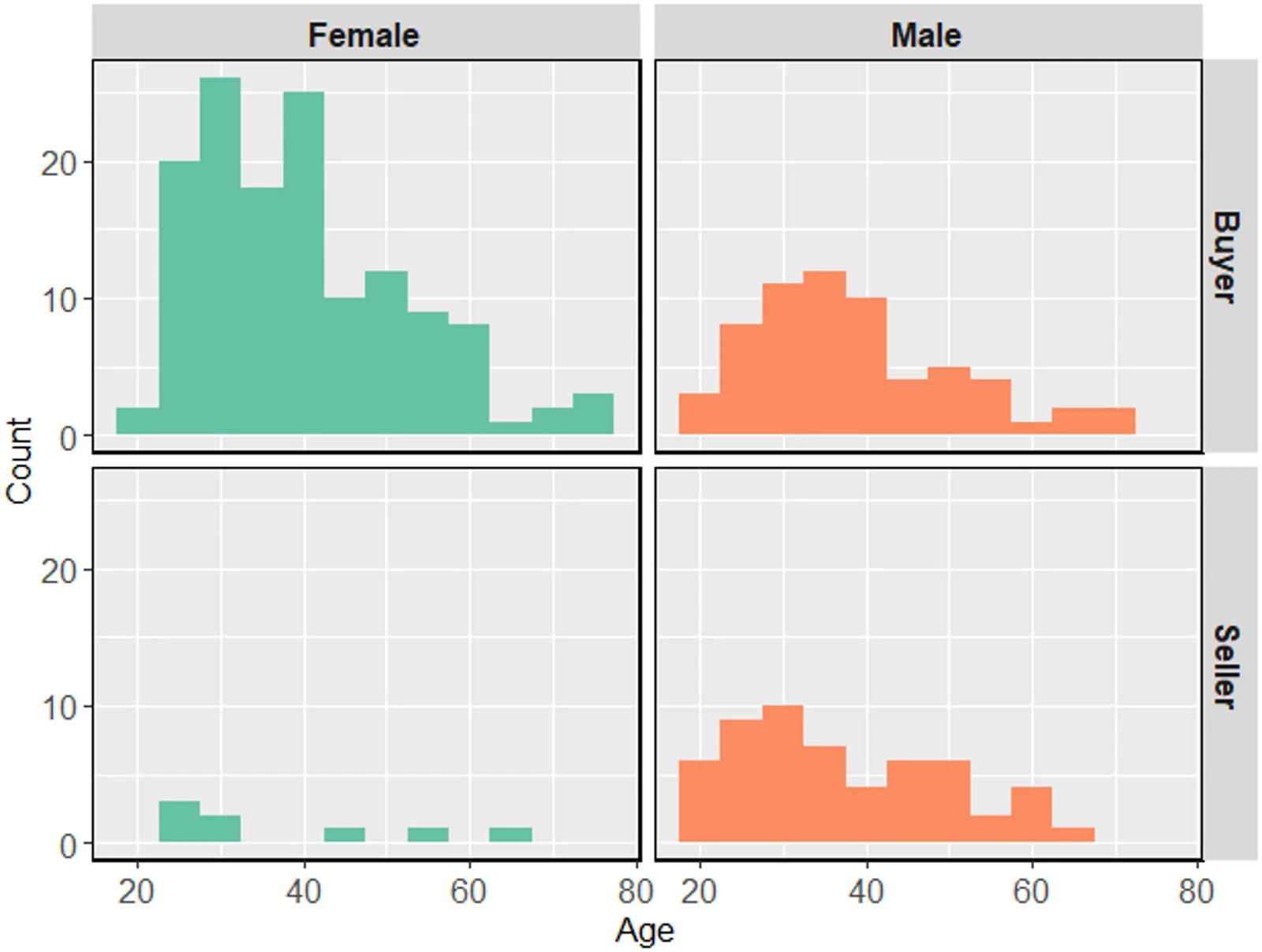
Age class distribution of stakeholders by gender (plant buyers and sellers: buyers are amateur gardeners, sellers include nursery owners and employees).

A total of 267 respondents (67 sellers and 200 buyers) were interviewed. Sellers included 28 nursery owners, 4 managers, and 35 employees. Among buyers, 68% (*n* = 136) were female and 32% (*n* = 64) were male (Fig. 2).

Among sellers, only one female was a nursery owner, and 7 of 35 employees were women. All respondents were over 18 years old; female buyers were predominantly aged 20 to 50, while male buyers were mostly under 40. No respondents identified as transgender. Most male sellers were under 40, with the largest group around age 30. Education levels varied: 40% held undergraduate and 41% postgraduate degrees. Among sellers, 61% had a school-level education and 27% were college graduates.

### Ornamental plants bought and sold in Bengaluru

**Fig. 3:**
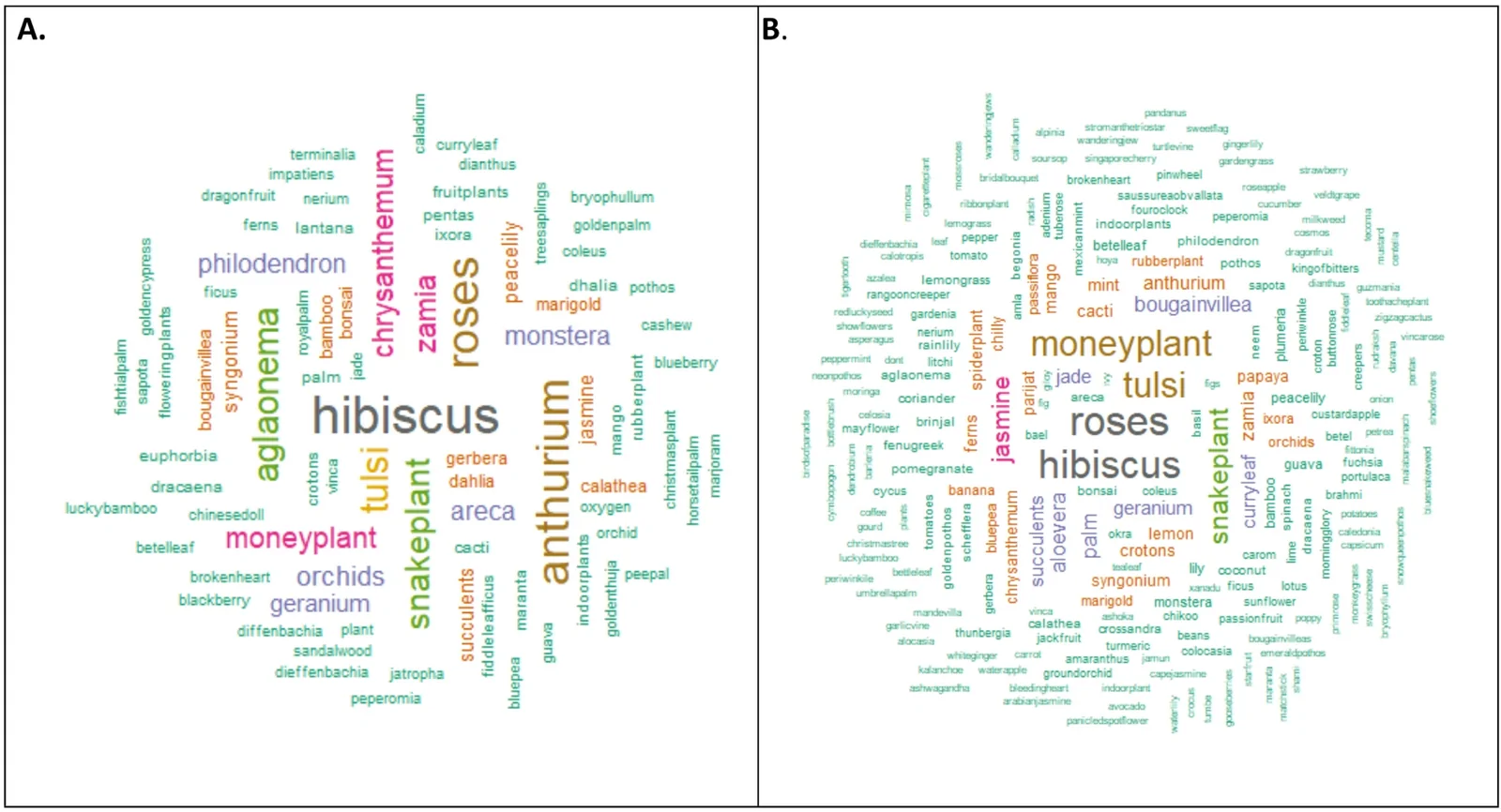
Word cloud representations of the most popular plant species grown by buyers (**A**) and as reported by sellers (**B**).

The most commonly cultivated plants (both alien and native) in homes, as reported by buyers, were the money plant (*Epipremnum aureum*), rose varieties (*Rosa* spp.), snake plant (*Dracaena trifasciata*), holy basil (*Ocimum tenuiflorum*), and hibiscus varieties (*Hibiscus* spp.). Similar trends were observed when sellers were asked about top-selling plants in their nurseries (Fig. 3).

Approximately 25% of respondents cited beauty as the primary reason for cultivating plants at home (Figure S1 in the Supplementary material). Additionally, 12% of respondents expressed a desire for greenery in their homes without a particular preference for plant type, while 12% mentioned the attractiveness of flowers. Nearly 9% of respondents cited religious or medicinal significance as the reason for growing plants. Practical factors, particularly ease of maintenance, which accounted for 17% of the responses, emerged as the primary consideration for selecting specific plants.

Jaccard similarity index analysis revealed no significant differences in the plants sold among nurseries in different administrative zones of the city (Table S1, Supplementary material). Zones such as Bommanahalli, Mahadevapura, and Rajarajeshwari Nagar, which host large nurseries, exhibit relatively high similarity among themselves (0.786 to 0.832). For the East and South zones, the Jaccard index of 0.583 indicates lower similarity in plant species compared to other areas. In the East zone, predominantly small nurseries were observed, in contrast to the densely clustered, large wholesale nurseries prevalent in the South zone.

### Plant disposal, species origin and IAS awareness

#### *Plant disposal methods*

**Fig. 4:**
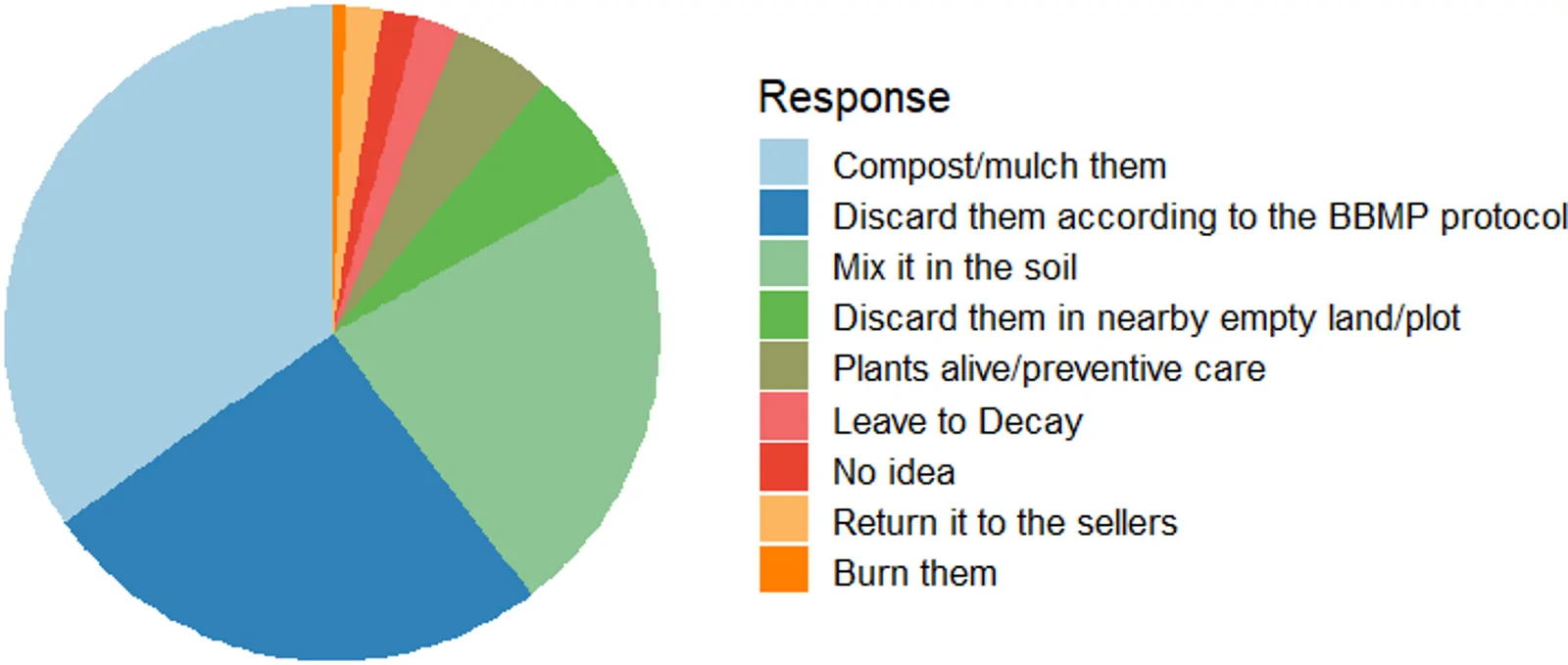
Relative proportions of different responses regarding how people choose to dispose of their ornamental plants.

When respondents were asked about the practice of disposing of plant waste, 35% mentioned composting or mulching it (Fig. 4).

Discarding according to the waste segregation protocol established by Bengaluru’s civic authority at the time of the study (i.e., separating wet and dry waste) is the second most favoured method, accounting for ~ 25% of responses, and mixing dead plant materials into the soil is another significant response (23%). Less common methods included discarding in nearby empty land or plots (6%), burning (1%) and leaving plant material to decay (2%). Additionally, 4% of respondents emphasised that they did not allow their plants to die and took proactive measures to ensure their survival.

#### *Familiarity with the origins of ornamental plants*

The photo-elicitation method highlighted the widespread recognition of the hibiscus (*Hibiscus × rosa-sinensis*) (Table 1). Other culturally significant plants - jasmine (*Jasminum auriculatum*), night-flowering jasmine *(Nyctanthes arbor-tristis*), and pinwheel flower (*Tabernaemontana divaricata*) were also commonly recognised. In contrast, the invasive lantana (*Lantana camara*), though common in Bengaluru, was rarely named despite being the second most familiar plant after hibiscus; 75% of buyers recognised it (Table 1). Other invasive alien species including common morning glory (*Ipomoea purpurea*), minnie root (*Ruellia tuberosa*), and bloodflower (*Asclepias curassavica*) were not recognised. The Z-test results revealed significant differences in the recognition of invasive species, compared to alien non-invasive and native species. For invasive versus alien non-invasive species, recognition proportions were 0.069 and 0.263, respectively (*p* < 2.2e-16). Similarly, for invasive versus native species, recognition proportions were 0.069 and 0.266, respectively (*p < 2.2e-16*).

Among nursery owners and sellers, a significant majority (~ 79%) reported selling lantana, indicating its widespread commercial availability (Table 1).

**Table 1:** Recognition of, and familiarity with, ornamental plants depicted in a photo collage shown to respondents as reported by the buyers and sellers.

Species name	Status in India	Buyers (*n* = 138)			Sellers (*n* = 66)	
		Recognised with name	Familiarity without name	Unrecognised	Sell	Do not sell
*Lantana camara* L.	Invasive	19	84	35	52	14
*Ipomoea purpurea* (L.) Roth	Invasive	22	24	92	43	23
*Thunbergia alata* Bojer ex Sims	Naturalised	2	8	128	24	42
*Tagetes erecta* L.	Cultivated	64	15	59	53	13
*Brugmansia suaveolens* (Humb. & Bonpl. ex Willd.) Sweet	Invasive	16	47	75	28	38
*Asclepias curassavica* L.	Invasive	2	22	114	26	40
*Bougainvillea spectabilis* Willd.	Naturalised	41	24	73	51	15
*Mirabilis jalapa* L.	Invasive	4	23	111	26	40
*Zephyranthes candida* Herb.	Cultivated	34	20	84	53	13
*Pelargonium × hybridum* (L.) L’Hér.	Cultivated	24	21	93	59	7
*Euphorbia milii* Des Moul.	Cultivated	9	46	83	45	21
*Hibiscus × rosa-sinensis* L.	Cultivated	94	28	16	61	5
*Cosmos bipinnatus* Cav.	Naturalised	11	12	115	33	33
*Nyctanthes arbor-tristis* L.	Cultivated	47	21	70	53	13
*Plumeria rubra* L.	Cultivated	48	56	34	58	8
*Jasminum auriculatum* Vahl	Cultivated	55	18	65	52	14
*Tithonia diversifolia* (Hemsl.) A.Gray	Invasive	1	36	101	21	45
*Ruellia tuberosa* L.	Invasive	3	27	108	29	37
*Nerium oleander* L.	Cultivated	32	42	64	52	14
*Tabernaemontana divaricata* (L.) R.Br. ex	Cultivated	13	33	92	41	25

However, only a small proportion (8%) demonstrated an understanding of its invasiveness. Approximately 5% of these sellers acknowledged offering hybrid lantana variants, which are claimed to be sterile and non-invasive. When questioned about the origin of various plants displayed in the collage, over 75% of buyers and more than 80% of sellers indicated no knowledge of the plants’ origins.

#### *Awareness about terms ‘invasive alien species’ or ‘invasive plants’*

**Fig. 5:**
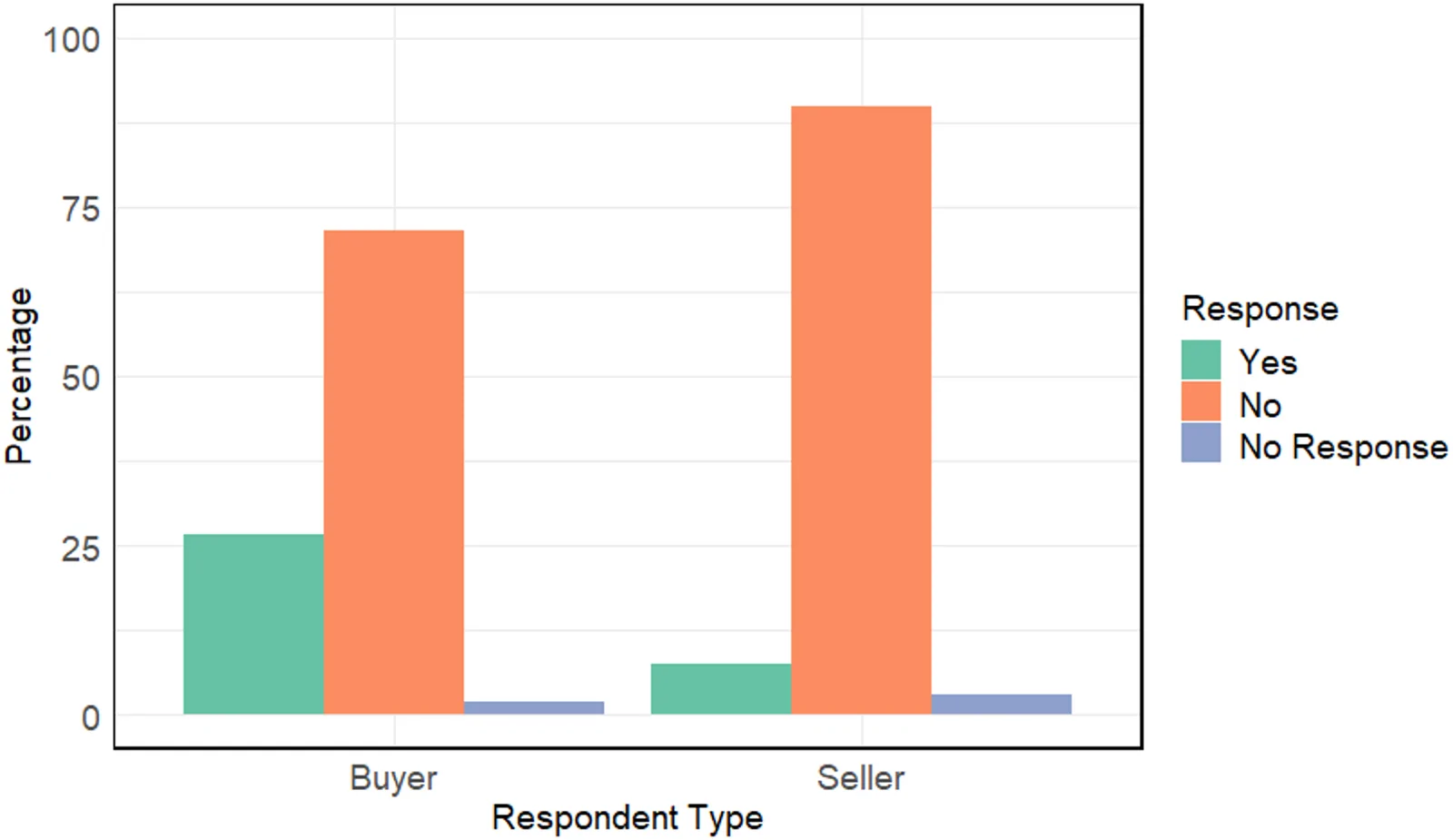
Proportion of respondents familiar with the term ‘invasive species’ among buyers and sellers.

Little awareness existed of the terms ‘invasive alien species’ or ‘invasive plants.’ Less than 30% of buyers were familiar with these terms, while only 5% of sellers reported familiarity with invasive species. A small percentage from both groups chose not to respond (Fig. 5).

A chi-square test revealed a significant association between stakeholder type and awareness of invasive species (*p* = 0.004), indicating buyers and sellers differ in awareness levels.

Awareness of IAS was best predicted by the model including age, gender, and highest qualification (Table 2; and Supplementary Table S2). The model showed that as age increased, likelihood of awareness of invasive species decreased marginally. Gender (male) was negatively related to awareness of invasive plants. Educational qualifications were positively related to awareness, with higher qualifications associated with greater likelihood of awareness of invasive species.

**Table 2:** The five best models of respondents’ awareness of invasive species in relation to demographic information and educational qualifications. Model selection was done using Akaike Information Criterion (AIC).

Models	Log Likelihoods	Delta AIC	AIC Weight
Response ~ Age + Gender + Highest.Qualification	−117.947	0.00	0.416
Response ~ Age + Highest.Qualification	−119.234	0.41	0.338
Response ~ Highest.Qualification	−121.320	2.44	0.123
Response ~ Gender + Highest.Qualification	−120.309	2.56	0.116
Response ~ Age + Experience + Highest.Qualification	−117.722	10.65	0.002

### Influence of awareness on buyer and seller behaviour

**Fig. 6:**
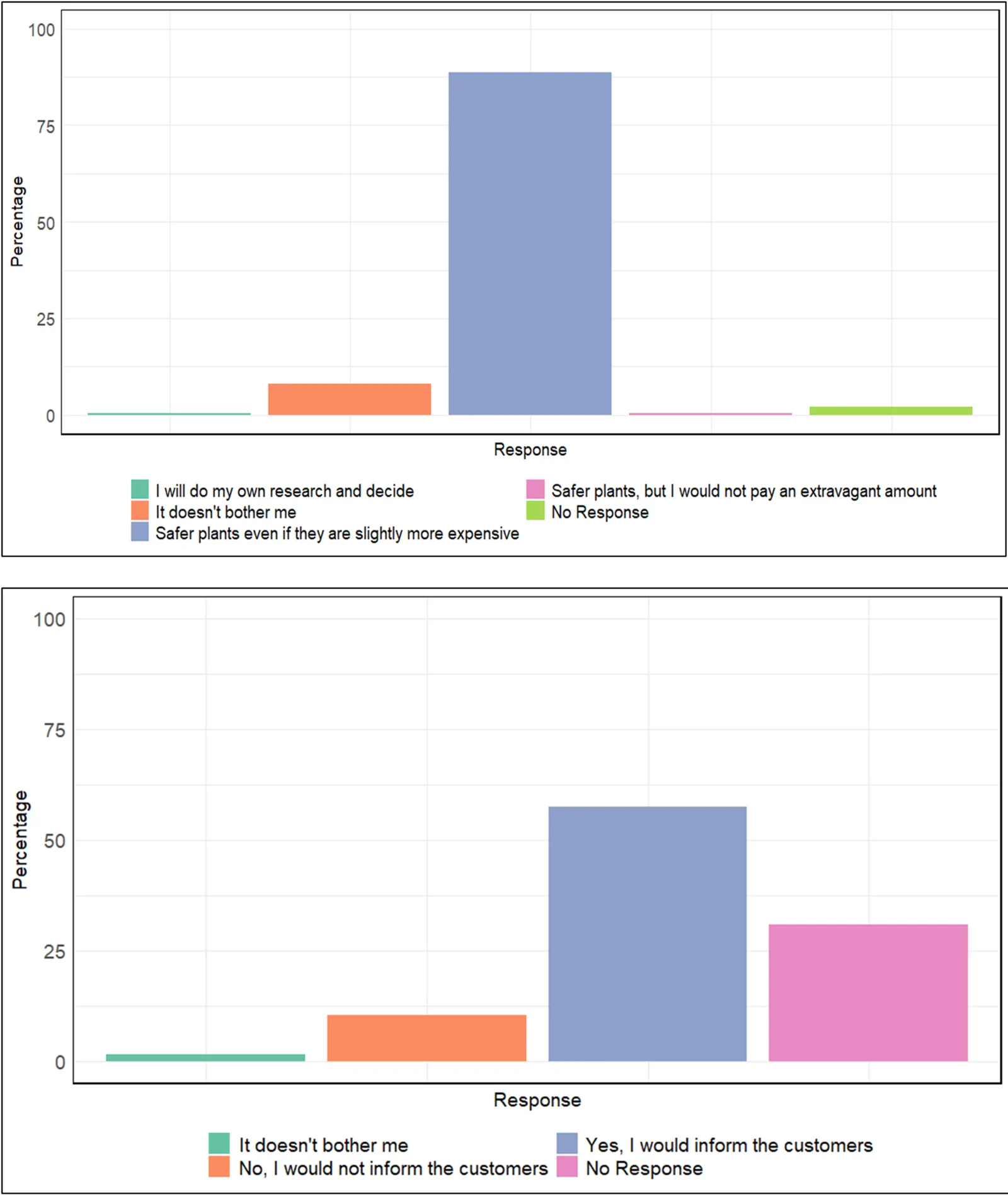
Informed buyers’ preference: choosing safer plants over invasive ones (A) and informed sellers’ response to inform customers’ about invasive/potentially invasive plants (B).

After being informed about invasive species and their potential impacts, buyers showed an inclination to reconsider their plant-purchase preferences. A significant majority (89%) expressed a preference for safer, albeit slightly more expensive, plants over cheaper invasive or potentially invasive varieties, although ~ 8% of buyers remained indifferent (Fig. 6).

When sellers were asked whether they would inform their customers about the invasive or potentially invasive nature of the plants they sold, after being informed of the subject, the majority (57%) responded that they would choose to inform their customers. Approximately 31% provided no response and a small percentage (10%) explicitly stated that they would not inform customers. Only a very small proportion (~ 2%) said that the issue did not concern them.

## Discussion

Invasion biology research shows global disparities, with urban ecosystems underrepresented relative to natural areas^18^. Urban areas, as centres for ornamental plant introduction and cultivation, serve as key points from which potential IAS can escape and spread into surrounding landscapes. While fewer than 10% of introduced ornamental horticultural plants escape cultivation, the large number cultivated makes this industry the main source of naturalised and invasive plants worldwide^4^. Most invasion biology research comes from the Global North; approximately 83% of invasive plant studies, drawn from 458 articles in Biological Invasions and NeoBiota (1999–2020), are from the Americas and Europe, with fewer from Hawaii, South Africa, Australia, New Zealand, and China^18^. The authors also emphasised that research in tropical regions remains limited, despite these areas being among the most biodiverse and threatened ecosystems. This imbalance highlights the need for broader research in urban and underrepresented regions. Our study addresses this gap by documenting the low awareness of IAS among ornamental plant buyers and sellers in Bengaluru, a major city in the Global South.

### Cultural, religious, and aesthetic factors influencing plant selection in Bengaluru

Our results showed that cultural, religious, and aesthetic factors shape plant choices in Bengaluru. This aligns with studies from Switzerland^33^ and China^8^, showing that aesthetic preferences often outweigh ecological concerns and increase the risk of spread of invasives. This creates a complex conflict between the crucial ecosystem services these plants provide such as climate regulation and cultural value^8,34^ and the collateral risk of biological invasions. In Bengaluru, hibiscus, holy basil, jasmine, and night-flowering jasmine are chosen for their cultural and religious value in daily rituals. Meanwhile, popular indoor plants such as snake plants and monsteras (*Monstera* spp.) are chosen for their attractive foliage and low maintenance, making them a good fit for high-density housing with limited outdoor space. This trend reflects the city’s shift from traditional gardens to high-rise apartments.

Plant choices appear linked to nursery size. Small nurseries favour plants with religious and cultural significance, such as holy basil, hibiscus, and chrysanthemum (*Chrysanthemum × morifolium*). The snake plant, marketed as an ‘oxygen plant’, is also popular for indoor air purification. Large nurseries stock more varieties, hybrids, and expensive plants, such as different types of orchids, bonsais, and less commonly available plants, such as calla lilies (*Zantedeschia aethiopica*) and azaleas (*Rhododendron* spp.), and are located on city outskirts with more space.

### Limited awareness of invasive species and their ecological impact

Consistent with the second hypothesis, the study identified a widespread lack of awareness regarding the origins and invasive potential of ornamental plants. Although aesthetics strongly influenced plant selection, most respondents were unfamiliar with the native or exotic status of the species, a pattern also observed in Brazil^35^. A study from Switzerland similarly found that many participants misidentified invasive species as native and were reluctant to manage them, primarily due to their attractive appearance^33^. This prioritisation of aesthetics over ecological awareness is evident in the present study, with many respondents unaware of plant origins, indicating widespread knowledge gaps. This is particularly concerning given that a significant proportion of plants sold online in India are non-native, with alien species outnumbering native species nearly threefold (Kesang Bhutia, unpublished data). Some of these non-native alien plants currently under cultivation may serve as sources for future invasive species, contributing to the phenomenon of invasion debt^36^. These seemingly harmless plants can eventually exhibit invasive behaviour, underscoring the necessity for proactive risk assessments within the ornamental plant industry.

Again, consistent with our second hypothesis, that public awareness of invasive species is limited, most respondents (both sellers and buyers) only demonstrated a superficial understanding of the concept. Invasive species were less recognised compared to alien non-invasive and native species. Although familiar with lantana, respondents did not recognise its invasive nature, simply referring to it as a “wild plant” commonly found in forests. The difficulty in identifying invasive ornamental plants, initially introduced for aesthetic reasons^7^, raises concerns about ongoing alien species introduction. These are often driven by the desire for novel and exotic flora but may unintentionally worsen ecological disruptions^37^. For instance, the book ‘Garden Flowers’ by Vishnu Swarup^38^, describing various plants commonly grown in Indian gardens at the time, lists several species that have since become invasive, such as *Ageratum conyzoides*,* Ageratum houstonianum*,* Celosia argentea*,* Mirabilis jalapa*,* Pontederia crassipes*,* Calceolaria mexicana*,* Ipomoea quamoclit*, and others. Many others, like *Catharanthus roseus*,* Cosmos bipinnatus*, and *Gomphrena globosa*, are now naturalised.

Our results indicate that awareness of invasive species is higher among younger and more educated individuals, suggesting it is a recent development concentrated in specific demographic groups. Individuals cited their familiarity with invasive species primarily from online articles and blogs, as well as through social media, newspapers and magazines. Leveraging these platforms could be important for reaching diverse audiences. Effective communication and educational campaigns are crucial, as emphasised by studies that highlight the role of the media and educational institutions in raising awareness and understanding of invasive species^31,39^.

Plant disposal practices emerged as another area of concern. While many respondents composted plant waste, an environmentally beneficial practice if done correctly, few recognised the risks posed by improper composting of invasive species. Effective composting requires temperatures high enough to destroy plant pathogens and weed seeds^40,41^. A considerable number of respondents also confessed to disposing of their plant waste by dumping it on nearby empty land or mixing it into the soil, believing it would cause no harm and might even improve soil fertility. This emphasises the need to promote responsible disposal practices and educate the public about the ecological impacts of gardening choices.

### The impact of awareness on attitudes and actions towards invasive species

Greater awareness of biological invasions increases support for protecting native species over invasive ones^42^ and promotes actions to prevent their spread^10^. This highlights the potential role of education and awareness in shaping consumer behaviour to back initiatives aimed at reducing the sales and spread of invasive species^43^. Our study showed that when respondents were informed about the ecological impacts of invasive species, their attitudes appeared to shift. While this could reflect genuine learning, social desirability bias (in which respondents give socially acceptable answers)^44^ may also play a role. Even accounting for this bias, the widespread willingness to pay slightly more in an anonymous, non-incentivised context suggests that many respondents may hold genuine environmentally conscious views, even if their actual purchasing behaviour is lower. Furthermore, our respondents were not learning about invasive species as an abstract concept; many had already experienced problematic plant behaviour in their own gardens, such as rapid spread or difficulty controlling them. Our intervention provided an ecological framework for connecting these first-hand observations to the concept of invasiveness. Research suggests that such experiential grounding creates stronger attitude-behaviour correlations than indirect learning^45^. Nevertheless, we acknowledge the attitude-behaviour gap where real-world constraints like price sensitivity may prevent stated intentions from manifesting as behavioural change and further research is needed to establish causal links between awareness and actual purchasing behaviour.

Educational interventions have been shown to be effective. For instance, a study in Portugal found that participants who had attended an IAS workshop a year earlier were better able to define and differentiate native, exotic, and invasive species, had greater awareness of IAS arrival in the country, and knew more about relevant legislation^46^. Similarly, Waliczek et al.^47^ found that college students who learned about invasive species developed more positive attitudes towards their management. Encouragingly, our results indicate a latent willingness among both buyers and sellers to adopt more environmentally responsible behaviours, thereby confirming our third hypothesis that informational interventions could influence stakeholder attitudes. For buyers, there is a clear need for targeted dissemination and outreach efforts to increase awareness of the ecological impacts of invasive species. Regarding sellers, small nurseries demonstrated a greater willingness to discuss the risks of invasive species with customers, whereas larger nurseries prohibited even the distribution of a brochure containing information on invasive ornamental plants (Supplementary material: D, E). This discrepancy may arise from the inherent conflict between educating customers about the potential consequences of invasive plants and safeguarding business interests.

### From gaps to actions: Improving awareness and policies on invasive ornamental plants

Despite the ecological threats posed by invasive ornamental plants, IAS awareness in the Global South remains under-researched. Of the 190 signatories to the Convention on Biological Diversity (CBD), only 17% (32 countries) have specific IAS legislation, most of them in the Global North^1,48^. South Asia, facing exceptionally high invasion risks^7,49^, has limited legal frameworks for IAS management. In India, existing national policies mostly focus on agricultural pests and weeds, with limited direct attention to broader invasive species management. This lack of consistent information mirrors issues in the United States, where disjointed regulations leave consumers and growers largely unaware of which species are invasive, leading to the ongoing purchase of harmful ornamentals^50^. To make matters worse, the existing regulations on the import of live plants and propagules in India remain antiquated. The Destructive Insects and Pests Act (1914), the Plants, Fruits, and Seeds (Regulation of Import into India) order (1989), and the Plant Quarantine (Regulation of Import into India) order (2003) provide a legislative framework to regulate the entry of insect pests and plant diseases from other countries. Under this framework, importing live plants, cuttings, bulbs, seeds, tubers, rhizomes, etc., requires a phytosanitary certificate and an import permit. Plant Quarantine (PQ) Order, 2003 contains twelve Schedules that detail how the provisions of the acts are being carried out (blob:https://pqms.cgg.gov.in/206771ee-4d55-45aa-a76a-4209eceea08b; accessed 21/12/2023). The list of quarantine plants (prohibited, restricted and regulated, Schedule VIII, amended in 2023) consists of 57 quarantine weed species, yet, only one invasive species, i.e., *Anthemis cotula* (Dog fennel), is listed as IAS in the Indian Alien Flora Information (ILORA) database^51^.

This regulatory gap is reflected in public awareness: our local study mirrors the national trend of limited knowledge about IAS. However, bridging this gap is essential for broader global goals; an integrated governance approach to biological invasions is fundamentally tied to achieving Sustainable Development Goal 11 (Sustainable Cities)^1^. In this study, the BBMP (now the GBA) was responsible for managing urban green spaces and regulating the disposal of plant waste. These responsibilities position the agency to effectively address IAS challenges. Rather than creating new governance systems, incorporating IAS awareness into current protocols could utilise existing infrastructure and administrative resources to mitigate invasion risks. Additionally, positioning IAS management as a central aspect of urban ecological resilience could underscore its significance to urban planners and municipal administrators, elevating it from a peripheral environmental concern to a priority within broader sustainability and climate adaptation strategies.

In California, the PlantRight program worked with retailers and customers to identify invasive ornamentals, promote alternatives, and raise awareness; reducing sales of listed invasive species from 44% to 20% over seven years^52^. Nurseries could attract more customers by selling non-invasive plants and adopting eco-friendly branding. Certification programs can offer a competitive advantage; for example, Australia’s ‘Gardening Responsibly Scheme’ provides a scientifically vetted list of low-risk species marked with its certified eco-label (gardeningresponsibly.org.au; accessed 19 July 2025). Only sellers who are registered participants in the ‘Gardening Responsibly Scheme’ are permitted to sell or distribute plants with this certification mark. Such programs can enhance a nursery’s reputation, provide marketing benefits, influence consumer preferences, and potentially increase sales^43^.

Teich et al.^53^ suggest that an opt-in labelling program created in partnership with sellers, governments, or non-profit organisations could serve as an incentive by providing buyers with information and potentially influencing their purchasing choices, without imposing regulatory costs. In our study, buyers were willing to pay slightly more for non-invasive, safer plants. By charging a premium for non-invasive plants, sellers could recoup the costs of the labelling program^53^. Sellers could also convey the potential of these species to become invasive, thereby fostering a more informed and responsible consumer base. However, given the inherent conflict of interest in which sellers may prioritise profits over ecological considerations, regulatory measures could play a pivotal role. Legislative interventions, such as restricting the inflow of potentially invasive species- a “blacklist” approach as adopted in South Africa, North America and in some European countries or strict import regulations as seen in New Zealand, which bans all alien species not already present unless approved by rigorous risk assessment could be helpful^7,36^.

Ensuring compliance with legislation and encouraging voluntary codes of conduct could be helpful. Hulme et al.^4^ suggest that mandatory industry codes of conduct be established, with consequences for non-compliance, to ensure that the industry takes responsibility for preventing the introduction and spread of invasive species. They further propose making pre-border risk assessments mandatory, along with post-border bans on the sale of these species, to further minimise their distribution and spread. Banerjee et al.^7^ also recommend imposing bans on the sale of established invasive species. They further suggest incentivising trade in native species to shift consumer preferences or making buyers aware of the potential impacts of their choices. Kumschick et al.^54^ observed that educating consumers about the ecological benefits of native plants reduces interest in alien species, as buyers generally prioritise environmental responsibility when given clear information.

### Conclusion

This study highlights the importance of improved awareness and management of invasive alien species (IAS) within the ornamental plant trade, particularly in rapidly urbanising areas of the Global South. In Bengaluru, India, both buyers and sellers demonstrated limited knowledge of IAS, with plant selection largely driven by cultural, religious, and aesthetic preferences that frequently overshadow ecological considerations. Awareness was positively associated with younger age and higher educational attainment, suggesting that demographically targeted outreach strategies could be especially effective. Encouragingly, when informed of IAS risks, a substantial majority of stakeholders expressed willingness to adopt more responsible purchasing and communication practices- indicating that knowledge gaps, once addressed, could potentially lead to a shift in attitudes and behavioural intentions, even if the translation of stated intentions into actual behaviour warrants further investigation.

Addressing these challenges requires a multifaceted approach: (1) strengthen public education and outreach to promote responsible purchasing and disposal practices concerning ornamental plants; (2) implement regulatory tools such as labelling schemes, stricter import controls, and risk assessments; and (3) foster collaboration among industry, urban governance bodies, and environmental organisations. As one of few empirical studies examining IAS awareness in the ornamental trade from the Global South, this work highlights the broader importance of expanding such research to other fast-growing cities. Bridging scientific knowledge and public awareness through these measures can meaningfully reduce the ecological risks posed by invasive ornamentals, while preserving the cultural and aesthetic values that urban gardening holds for communities like Bengaluru.

## Supplementary Information

Below is the link to the electronic supplementary material.

Supplementary Material 1

## Data Availability

The interview data for this study are not publicly available at this time because they are part of an ongoing PhD thesis. The data will be deposited in a public repository following the formal submission and defense of the thesis. In the meantime, they are available from the corresponding author upon reasonable request.
