# Supplementary material for: Assessing awareness to aid action: Stakeholders’ perception of invasive alien species in a metropolis from the Global South
Source: Sci Rep. 2026 Aug 26;16:26805. doi: 10.1038/s41598-026-59488-6 (PMC13518948; doi:10.1038/s41598-026-59488-6)
Supplement: Supplementary file 1 [file 41598_2026_59488_MOESM1_ESM.pdf]

Supplementary Materials

A: Questionnaire for buyers

Interviewer: Date:

Name and address of the Nursery:

Section A: Personal Demographics Survey

Name:	*Age:
*Gender:	Occupation:
*Highest Qualification:	Contact No:
*Locality:	Email-ID:

**Mandatory*

Section B: What & why

1. **Do you have a garden/plants at home?**
   If yes, move to question 2
   If no, move to question 3

2. **What plants do you have in your garden/home?**

3. **Will you be purchasing any plants today?**
   If yes, move to question 4
   If no, move to question 6

4. **What plants are you planning to buy today?**

5. **Why did you choose to buy these plants?**

6. **How do you dispose of your plants?**

Section C: Online Nursery

7. **Have you ever bought any ornamental plants/seeds/saplings online?**
   If yes, ask them the names.
   If no, then move to question 8

8. **What is/are the reasons to buy plants from an online nursery?**

<u>Section D: Pandemic influence</u>

9. **For how long you have been gardening?**
<2 years, 2-5 years, 5-10 years, 10-15years, 15-20years. >20 years

10. **Could you kindly explain shortly how the pandemic has influenced your gardening hobby?**

<u>Section E: Photo-Elicitation method</u>

11. **Are you familiar with any of the species shown here in the pictures?**
If yes: Ask them to point out those species. Then move to question 11
If no: Move to question 12

12. What is the source of familiarity of these species?

<u>Section F: Idea & Knowledge</u>

13. Are you aware of where some of these ornamental plants come from?

If yes, request them to give an example
If no, then move to question 13

14. Have you ever seen flowering plants spread over a large area?
If yes, request them to briefly explain

15. Have you heard the term 'Invasive Species'?
If yes, ask from where and request to briefly explain it. Move to question 15
Newspapers___ Magazines___ Television___ Internet___ Other____
If no, then conclude the interview.

16. Can you give me the names of some invasive species in India that you are aware of?
If not in India, then from anywhere

17. If you are given an option of buying inexpensive but invasive ornamental species and slightly expensive but safer species, which one will you choose to buy?

B: Questionnaire for Sellers

Interviewer: Date:

Name and address of the Nursery:

Section A: Personal Demographics Survey

Name:	*Age:
*Gender:	*Designation:
*Highest Qualification:	Contact No:
*Locality:	Email-ID:

**Mandatory*

1. **What are the most popular plants in your nursery?**

2. **Why do you think people prefer these plants over others?**

3. **From where do you source your plants?**

4. **Do people ask you for a recommendation of plants?**
   If yes, ask how often they get asked and the names of plants they usually recommend, and why.

5. **How do you dispose of your plants?**

6. **Do you sell plants online? (tie-ups with e-commerce platforms or own website)**
   If yes, ask why and when did they start.

Section D: Pandemic influence

7. **For how long you have been in this business?**
   <2 years, 2-5 years, 5-10 years, 10-15years, 15-20years. >20 years

8. **Could you kindly explain shortly how the pandemic has influenced your business?**

Section E: Photo-Elicitation method

9. **Do you sell any of the species shown here in the pictures?**

If yes: Ask them to point out those species.

Section F: Idea & Knowledge

10. **Are you aware of where some of these ornamental plants come from?**
    If yes, request them to give an example
    If no, then move to the next question

11. **Have you ever seen flowering plants spread over a large area?**
    If yes, request them to briefly explain

12. **Have you heard the term 'Invasive Species'?**
    If yes, ask from where and request to briefly explain it. Move to question 15
    Newspapers___ Magazines___ Television___ Internet___ Other____
    If no, then conclude the interview.

13. **Can you give me the names of some invasive species in India that you are aware of?**
    If not in India, then from anywhere

14. **If you are given an option, would you be willing to inform buyers of invasive, potentially invasive, and safe ornamental plants?**

C: Collage for the Photo-Elicitation method

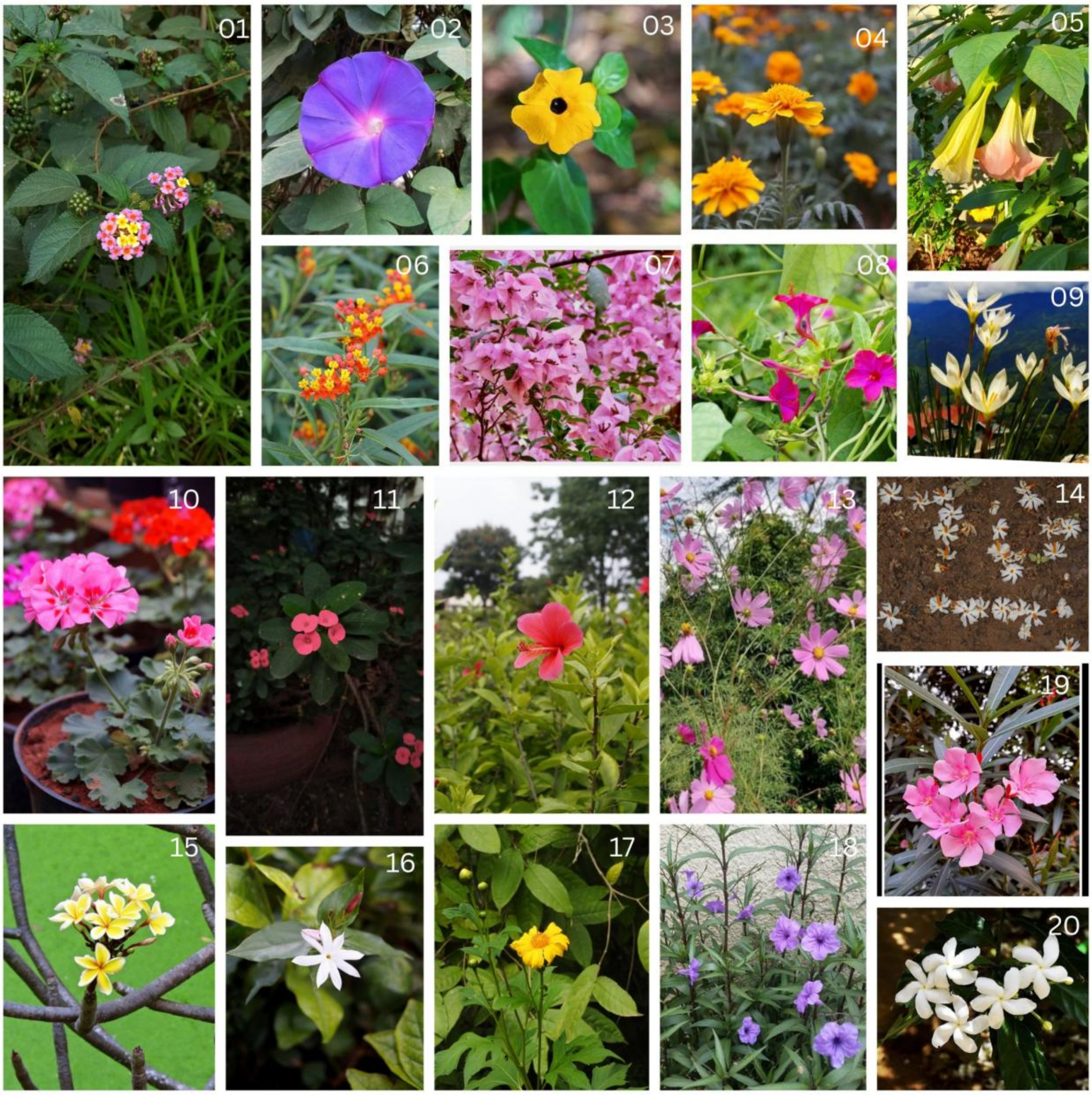

D: Brochure in English

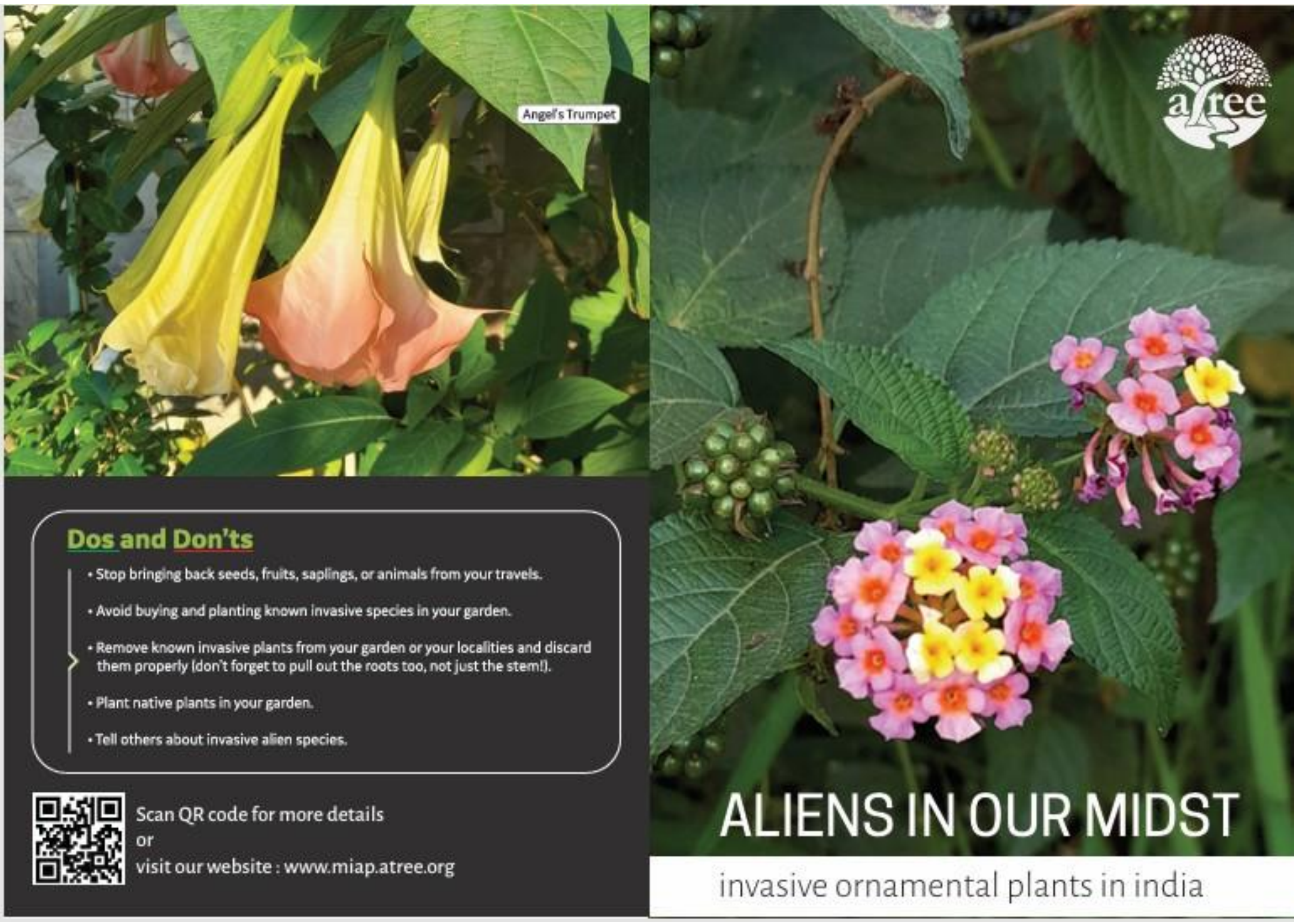

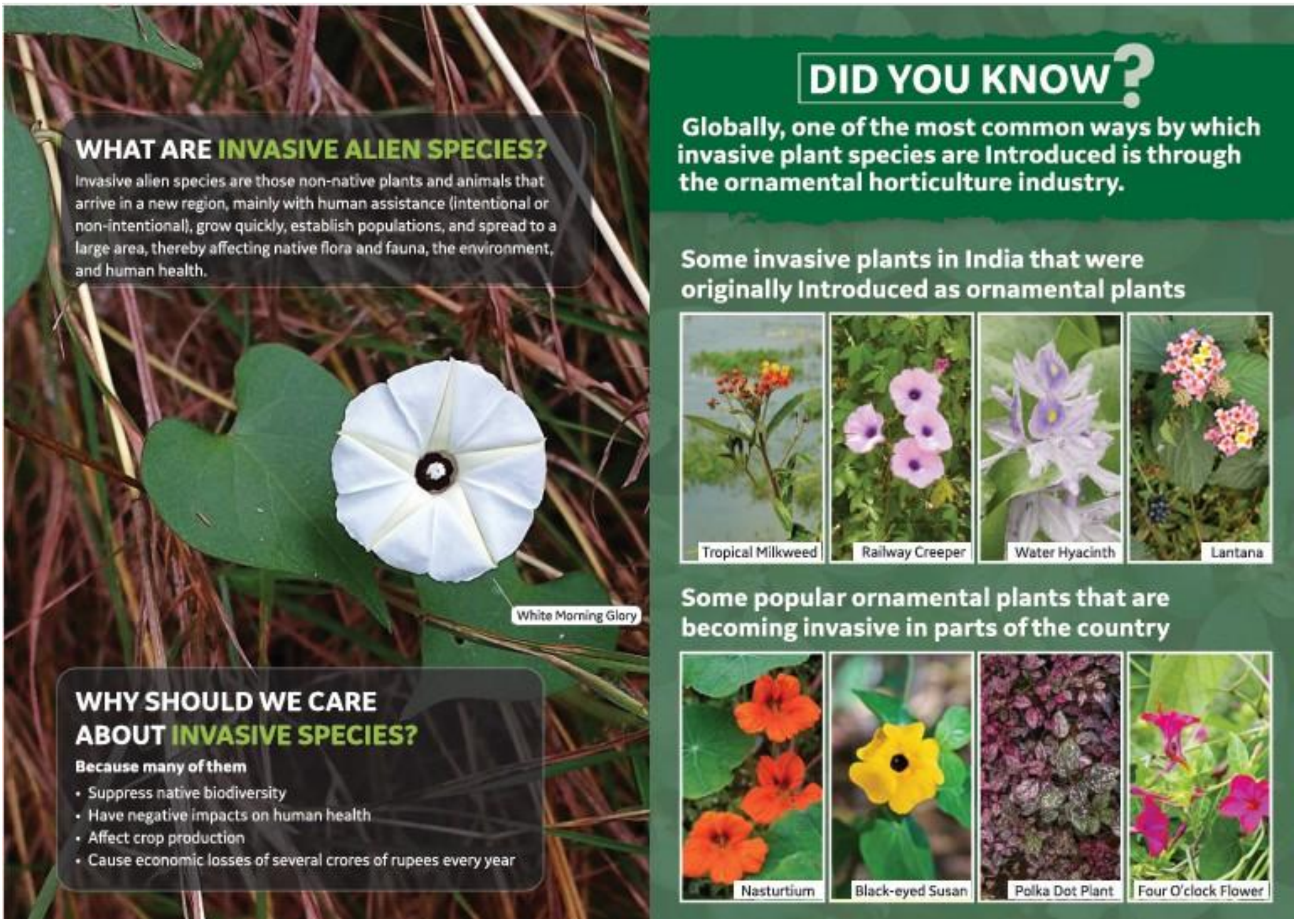

E: Brochure in Kannada

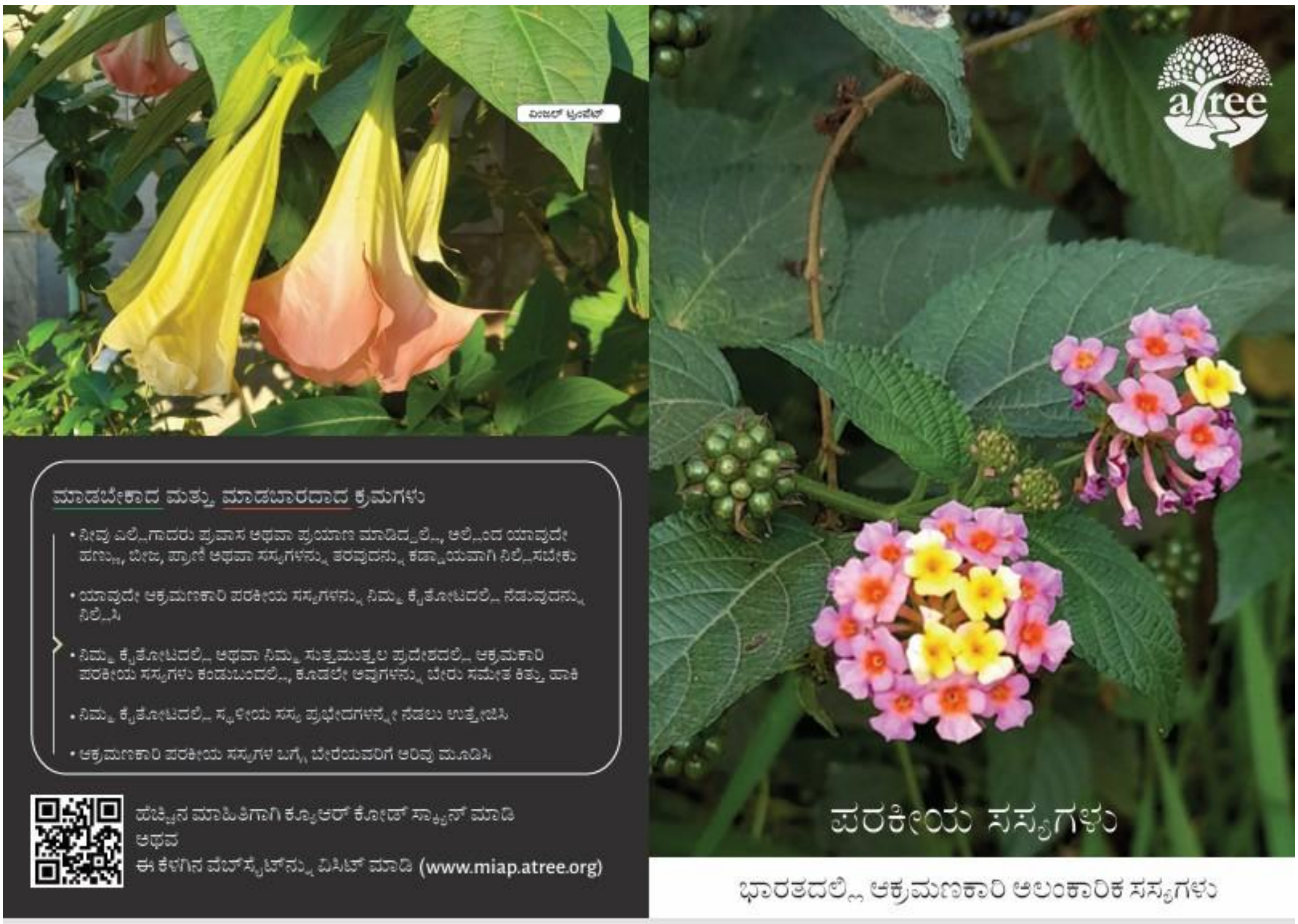

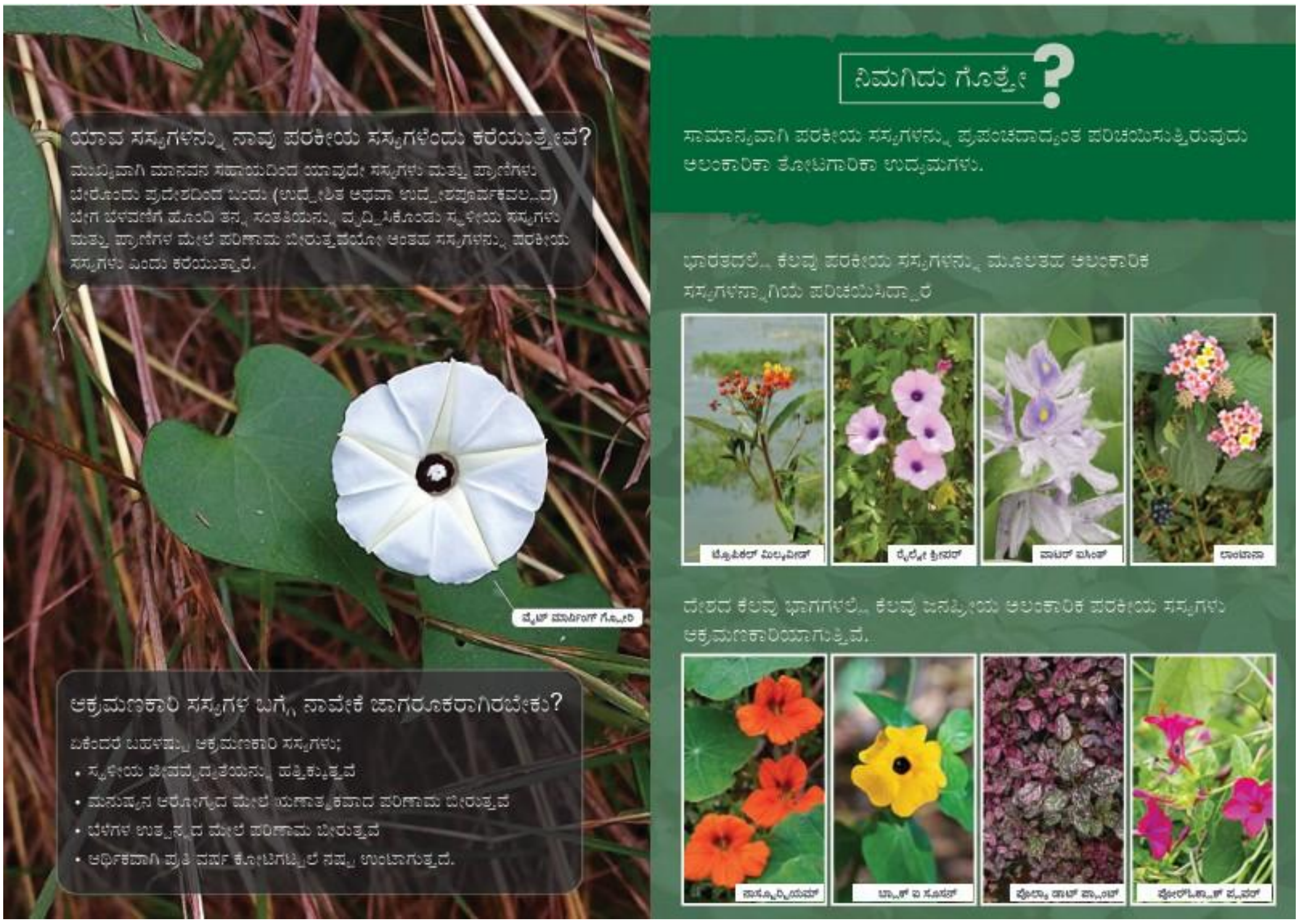

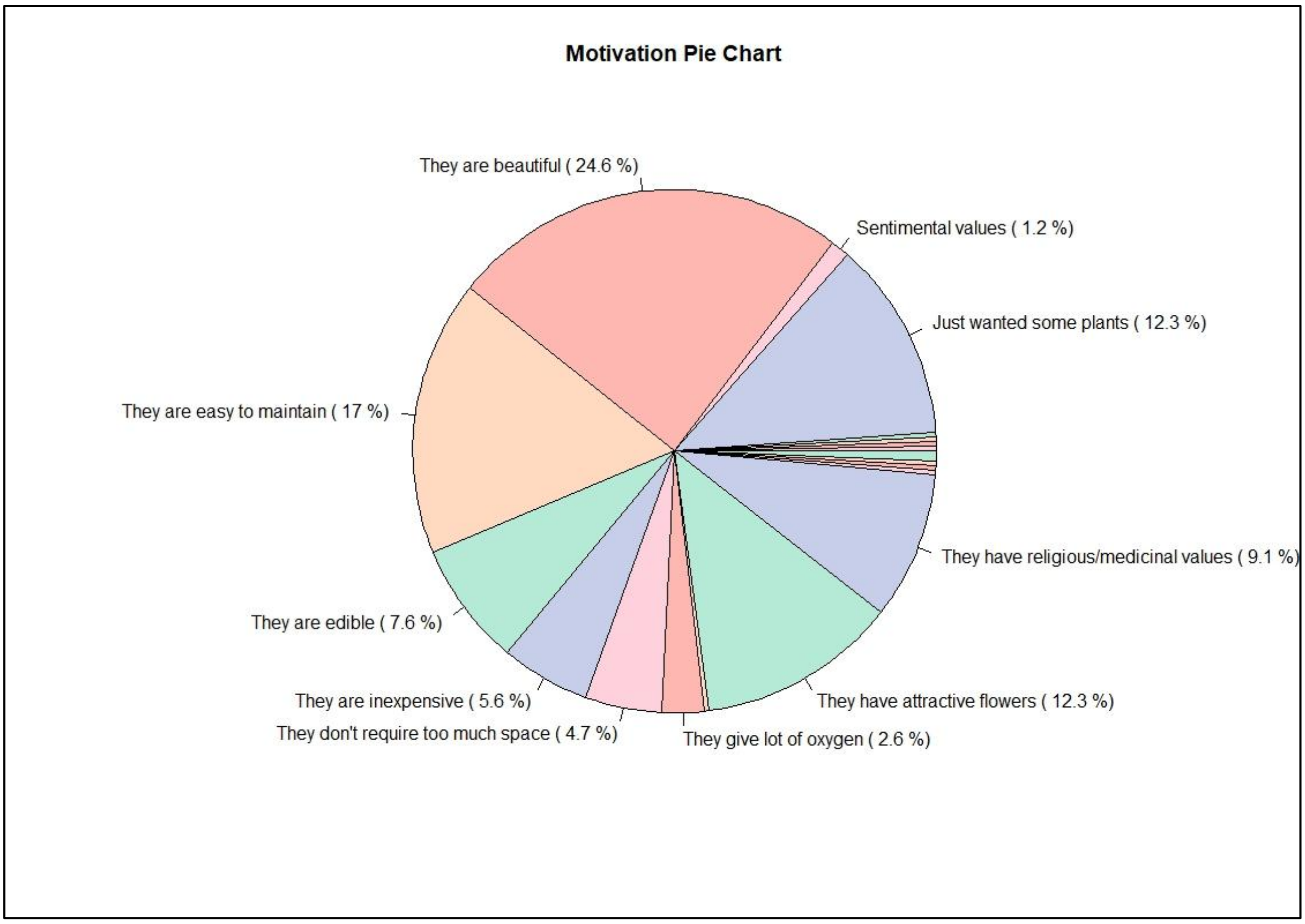

Fig S1: Survey responses regarding people's motivation for having plants at home. (Reasons mentioned by fewer than 1% of respondents are not labelled.)

Table S1. Comparative analysis of plant species sold in nurseries located in different administrative zones (See Figure 1 for a map depicting the location of the administrative zones mentioned here) of Bengaluru, revealing patterns of similarity and dissimilarity using Jaccard similarity index.

Zones → ↓	Bommanahalli	Dasarahalli	East	Mahadevapura	RR Nagar	South	West	Yelahanka
Bommanahalli	1.000	0.693	0.646	0.832	0.786	0.773	0.670	0.752
Dasarahalli	0.693	1.000	0.775	0.651	0.750	0.652	0.759	0.658
East	0.646	0.775	1.000	0.621	0.738	0.583	0.816	0.600
Mahadevapura	0.832	0.651	0.621	1.000	0.738	0.792	0.660	0.786
RR Nagar	0.786	0.750	0.738	0.738	1.000	0.687	0.744	0.694
South	0.773	0.652	0.583	0.792	0.687	1.000	0.603	0.898
West	0.670	0.759	0.816	0.660	0.744	0.603	1.000	0.651
Yelahanka	0.752	0.658	0.600	0.786	0.694	0.898	0.651	1.000

Table S2. Results of a logistic regression analysis to determine the best predictors of knowledge about invasive alien species. Models are ranked by their Delta AIC value; the best model is highlighted in bold.

Model no	Models	Log Likelihoods	Delta AIC	AIC Weight
14	**Response ~ Age + Gender + Highest.Qualification**	-117.947	0.00	0.416
10	Response ~ Age + Highest.Qualification	-119.234	0.41	0.338
9	Response ~ Highest.Qualification	-121.320	2.44	0.123
13	Response ~ Gender + Highest.Qualification	-120.309	2.56	0.116
12	Response ~ Age + Experience + Highest.Qualification	-117.722	10.65	0.002
6	Response ~ Age + Gender	-129.805	11.02	0.002
16	Response ~ Age + Gender + Experience + Highest.Qualification	-116.809	11.10	0.002
5	Response ~ Gender	-131.386	12.14	0.001
11	Response ~ Experience + Highest.Qualification	-119.891	12.73	0.001
15	Response ~ Experience + Gender + Highest.Qualification	-119.061	13.33	0.001
2	Response ~ Age	-133.945	17.25	0.000
1	Response ~ 1	-135.162	17.65	0.000
7	Response ~ Experience + Gender	-129.823	21.59	0.000
8	Response ~ Age + Experience + Gender	-128.993	22.09	0.000
3	Response ~ Experience	-133.608	27.02	0.000
4	Response ~ Age + Experience	-132.968	27.88	0.000
